# Development of Recommendations for the Digital Sharing of Notes With Adolescents in Mental Health Care: Delphi Study

**DOI:** 10.2196/57965

**Published:** 2024-06-06

**Authors:** Martine Stecher Nielsen, Aslak Steinsbekk, Torunn Hatlen Nøst

**Affiliations:** 1 Department of Mental Health, Faculty of Medicine and Health Sciences, Norwegian University of Science and Technology, Trondheim, Norway; 2 Department of Public Health and Nursing, Faculty of Medicine and Health Sciences, Norwegian University of Science and Technology, Trondheim, Norway; 3 Norwegian Centre for E-health Research, Tromsø, Norway; 4 Clinic of Anaesthesia and Intensive Care, Clinical Research Facility, St. Olavs hospital, Trondheim, Norway

**Keywords:** electronic health record, EHR, electronic health records, EHRs, electronic medical record, EMR, electronic medical records, EMRs, patient record, health record, health records, personal health record, PHR, online access to electronic health records, open notes, clinical notes, adolescent mental health care, adolescent mental health, child mental health, mental health, mental illness, mental illnesses, mental disorder, mental disorders, recommendations, Delphi study, digital mental health, e-health, eHealth, e–mental health, health care professionals, digital health care

## Abstract

**Background:**

In many countries, health care professionals are legally obliged to share information from electronic health records with patients. However, concerns have been raised regarding the sharing of notes with adolescents in mental health care, and health care professionals have called for recommendations to guide this practice.

**Objective:**

The aim was to reach a consensus among authors of scientific papers on recommendations for health care professionals’ digital sharing of notes with adolescents in mental health care and to investigate whether staff at child and adolescent specialist mental health care clinics agreed with the recommendations.

**Methods:**

A Delphi study was conducted with authors of scientific papers to reach a consensus on recommendations. The process of making the recommendations involved three steps. First, scientific papers meeting the eligibility criteria were identified through a PubMed search where the references were screened. Second, the results from the included papers were coded and transformed into recommendations in an iterative process. Third, the authors of the included papers were asked to provide feedback and consider their agreement with each of the suggested recommendations in two rounds. After the Delphi process, a cross-sectional study was conducted among staff at specialist child and adolescent mental health care clinics to assess whether they agreed with the recommendations that reached a consensus.

**Results:**

Of the 84 invited authors, 27 responded. A consensus was reached on 17 recommendations on areas related to digital sharing of notes with adolescents in mental health care. The recommendations considered how to introduce digital access to notes, write notes, and support health care professionals, and when to withhold notes. Of the 41 staff members at child and adolescent specialist mental health care clinics, 60% or more agreed with the 17 recommendations. No consensus was reached regarding the age at which adolescents should receive digital access to their notes and the timing of digitally sharing notes with parents.

**Conclusions:**

A total of 17 recommendations related to key aspects of health care professionals’ digital sharing of notes with adolescents in mental health care achieved consensus. Health care professionals can use these recommendations to guide their practice of sharing notes with adolescents in mental health care. However, the effects and experiences of following these recommendations should be tested in clinical practice.

## Introduction

In many countries, health care professionals are legally obligated to share information from electronic health records (EHRs), including clinical notes, medications, and test results with patients [1,2]. This information is often shared through patient portals and aligns with the growing focus on patient-centered care and patient engagement to improve health care services and individual health outcomes, such as quality of life and mental health status [3,4]. However, this practice may pose challenges for health care professionals working with adolescents in mental health care, such as preventing potential harm arising from accessing mental health notes or limiting adolescents’ confidentiality [5,6].

Although health care professionals’ experience of sharing notes with adolescents in mental health care has not been studied, health care professionals in adult mental health care have expressed concerns about the sensitive nature of notes in mental health care and whether reading them can be harmful to the patient or damaging for the therapeutic relationship [7-9].

Moreover, it has been stated that different levels of autonomy and maturity among adolescents can pose challenges regarding the consequences of having access to notes about themselves [6,10]. Additionally, the possibility of parents or guardians accessing notes meant for the adolescent can compromise the confidentiality of what is discussed between the health care professional and the adolescent and impede the adolescent’s autonomy [5,10,11]. These potential harms make it challenging for health care professionals to determine what type of information from the EHR should be shared and when [6,12].

Health care professionals have called for recommendations and support on how to handle challenges with sharing notes with adolescents in mental health care or their guardians [8,10]. The World Health Organization has proposed that recommendations should be based on formal consensus methods such as the Delphi method [13]. Over the last decade, such recommendations have been made in related areas, for example, by providing guidance on supporting self-management and the transition to adult health care for adolescents with chronic somatic diseases [14-16] and communicating with young people in mental health care about their online behavior [17]. Such recommendations for health care professionals typically include providing adolescents and young people with information about the specific topic or treatment program early on and relevant topics to cover when giving information [14-17]. While these recommendations are often either created based on or evaluated by members of medical societies and associations, the views of diverse staff members working in the specific field are not always included, potentially excluding some experiences [16,18]

To our knowledge, no recommendations are available to guide health care professionals’ digital sharing of notes with adolescents in mental health care despite the specific challenges associated with this practice. Therefore, the aim was to reach a consensus among authors of scientific papers on recommendations for health care professionals’ digital sharing of notes with adolescents in mental health care and to investigate whether staff at child and adolescent specialist mental health care clinics agreed with the recommendations.

## Methods

To address the aims, a Delphi study and a cross-sectional study were conducted. The CREDES (Guidance on Conducting and Reporting Delphi Studies) [19] and STROBE (Strengthening the Reporting of Observational Studies in Epidemiology) guidelines for cross-sectional studies [20] were consulted during the planning and reporting of the study.

### Delphi Study With Authors of Scientific Papers

The Delphi method [21] was used to reach a consensus on recommendations for the digital sharing of notes with adolescents in mental health care. The Delphi study involved creating suggestions for recommendations, receiving feedback on the recommendations from Delphi participants, and creating final recommendations based on consensus. A literature search was performed to develop suggestions for recommendations and to identify participants for the Delphi study.

#### Literature Search

The aim was to include scientific papers indexed in PubMed about the digital sharing of clinical notes with adolescents in general, somatic, and mental health care, and adults in mental health care. Moreover, relevant papers identified in the references of the papers from the PubMed search were invited to participate. The search for papers was performed in PubMed in April 2023 by searching the references of the papers meeting the eligibility criteria (Table 1).

The publication date range was selected because the field is rapidly evolving, and most research relevant to our aim has been conducted within the last couple of years. An initial review of older references showed that the digital solutions, particularly those from before 2016, differed from later publications (eg, by being prototypes made for the research project). Moreover, they were, to a greater extent, considering perceptions and expectations toward potential digital solutions. Peer review in international journals indexed in PubMed was used as a proxy for quality, and hence, authors of protocols, unpublished papers, and gray literature were excluded due to the challenge of evaluating their quality.

**Table 1.** Inclusion and exclusion criteria for identifying relevant publications.

Area		Inclusion criteria	Exclusion criteria
**Criteria**			
	Population	Adolescents (in general) Adolescents in psychiatry/mental health care Adolescents in somatic health care Adults in psychiatry/mental health care	All other study populations
	Phenomenon of interest	Electronic health record Patient-accessible electronic health record Access to clinical notes Patient portal use	All other phenomena
**Information sources and methods**			
	Identifications of papers via database: database name, search string, and time period	Relevant papers from PubMed search: “(((Adolescent[MeSH Terms]) OR (Mental Health[MeSH Terms])) OR (Psychiatr*[MeSH Terms])) AND (electronic health record*[MeSH Terms])” from 2021 to 2023	Papers from the PubMed search published before 2021
	Identification of papers via other methods: search method and time period	Relevant references from the papers identified in the PubMed search 2016‐2020	Papers published before 2016
**Limits and restrictions**			
	Language	English	Languages other than English
	Type of publications	Papers from empirical studies and reviews published in peer-reviewed journals	Gray literature, protocols, and unpublished papers

#### Participants

All authors of the scientific papers identified in the literature search were invited to participate in the Delphi study if their email addresses could be identified through the publication, their institutions’ web pages, or a Google search.

Together with the invitation to participate, the authors received a description of the task and the project’s purpose. Additionally, they received information explaining how they had been selected, why their participation was requested, what participation involved, and details regarding the storage and use of their data.

#### Data Collection

The Delphi study started with screening the scientific papers included from the literature search to identify findings that could be turned into suggestions for recommendations (Multimedia Appendix 1 [7-10,22-53]). This process is further reported in the Analysis and Results sections.

The authors who created the suggestions have experience from research on patient portals, adolescents, and mental health. Care was taken to ensure that the created suggestions were based on the reviewed literature. Before sending out the suggested recommendations, they were discussed with a research group at a university that possesses expertise in research related to digital health, patient participation, and patient education. The suggestions for recommendations were sent to the Delphi participants between June and September 2023.

The Delphi participants’ responses to the recommendations were collected in two rounds, referred to as round 1 and round 2. Due to the summer holiday, the Delphi participants were given 8 weeks to respond to round 1 and 6 weeks in round 2. Two reminders were sent 2 weeks apart for both rounds.

In both rounds, the Delphi participants received an email with a link to a web-based questionnaire with the suggested recommendations for the digital sharing of notes with adolescents in mental health care. Adolescence was defined as being legally old enough to access their notes digitally. The participants were asked to score their agreement with each recommendation on a Likert scale from 1 to 5 (“strongly agree,” “agree,” “don’t know,” “disagree,” and “strongly disagree”). In round 1, the participants could also comment on each recommendation and give overall comments (Multimedia Appendix 2).

In round 2 of the Delphi study, participants who responded in round 1 were asked to score the recommendations supported in round 1 (in either their original or a modified form based on comments) and the recommendations generated based on comments in round 1. Moreover, the participants were informed about the proportion of participants who agreed with each of these recommendations in round 1, in accordance with recommendations for Delphi studies [19,54-56]. For both rounds, demographic data were collected on participants’ gender, profession, age, and years of working experience.

#### Analysis

The process of making the recommendations involved three steps: identifying scientific papers, creating suggestions for recommendations, and receiving feedback from the Delphi participants. In the first step, the eligibility criteria were first used to screen the titles and the abstracts, and then the full paper of those not possible to classify based on the title or abstract. Finally, the references of the included papers were screened to identify additional relevant studies.

The second step involved creating suggestions for recommendations. The results of all scientific papers were first coded in NVivo 14 (Lumivero) by the first author, who identified codes related to experiences, effects, views, and expectations regarding access to EHRs. After this, the coded results were discussed with the other authors who read the coded results and some of the papers. The authors prepared the recommendations in an iterative process, where several discussions were held on how the coded results could be transformed into recommendations. The first author proposed suggestions, which were then discussed until there was agreement among all authors that the suggested recommendations accurately represented the coded results. Unambiguous results from the papers were used to suggest recommendations, while conflicting or hesitant results were used to propose mutually exclusive recommendations.

The third step involved receiving feedback on the recommendations from the Delphi participants, where the scoring and comments were considered. A predetermined criterion for agreement was set for both rounds; a consensus was reached if 70% of participants responded with “strongly agree” or “agree” with the recommendation [54,55]. The recommendations that did not achieve consensus were dropped unless comments suggested modifications. The authors reviewed all the free-text comments and incorporated suggestions from comments that proposed alternative formulations of recommendations or suggested new recommendations for the preparation of round 2. The authors considered the wording of the recommendations, achieving consensus and suggestions for new recommendations in several meetings and rounds of review before round 2 was sent out. Answers to round 2 from authors who were health care professionals and those who were not were compared in a subgroup analysis to determine potential differences in the proportions agreeing. The responses from round 2 were used to create the final list of recommendations.

### Cross-Sectional Study With Staff

A cross-sectional study was used to investigate if staff at specialist child and adolescent mental health care clinics agreed with the recommendations for the digital sharing of notes with adolescents in mental health care. For this purpose, a survey with a web-based questionnaire was conducted in October 2023.

#### Participants

Participants in the cross-sectional study were staff working at child and adolescent specialist mental health care clinics geographically spread in Norway. Inclusion criteria were staff at child and adolescent specialist mental health care clinics who had contact with patients, including administrative staff. This choice was made because different types of staff can provide information about digital access to mental health notes at various times. Staff without patient contact, such as cleaning personnel, were excluded.

To recruit staff, four departments of child and adolescent mental health at university hospitals, geographically spread in Norway, were asked to distribute invitations to relevant clinics in their region. The total number of staff at their affiliated clinics was approximately 690. Eligible staff received information about the study and a link to the questionnaire.

#### Data Collection

The questionnaire sent to the staff at the specialist child and adolescent mental health care clinics included the recommendations that achieved consensus in the Delphi process translated to Norwegian. Moreover, questions about their gender, years of work experience in child and adolescent specialist mental health care, profession, and role at the clinic were asked. The staff were informed about the origin of the recommendations and instructed to think about adolescents as legally old enough to access their clinical notes digitally. For each recommendation, they were asked whether they “agreed” or “disagreed.” The link for the questionnaire was open for 1 month.

#### Analysis

To investigate whether staff at child and adolescent specialist mental health care clinics agreed with the recommendations developed in the Delphi study, the frequencies of agreement and disagreement with each statement were calculated. Moreover, descriptive statistics were performed on the sociodemographic data to present the characteristics of the staff.

### Ethical Considerations

This study was approved by the Norwegian Agency for Shared Services in Education and Research (717040), which ensures that the data processing is performed in accordance with national data protection legislation. All methods were carried out in accordance with the Declaration of Helsinki. Informed consent was obtained from all participants. None of the participants received any compensation for their participation.

## Results

### Results From the Delphi Study

**Figure 1.**
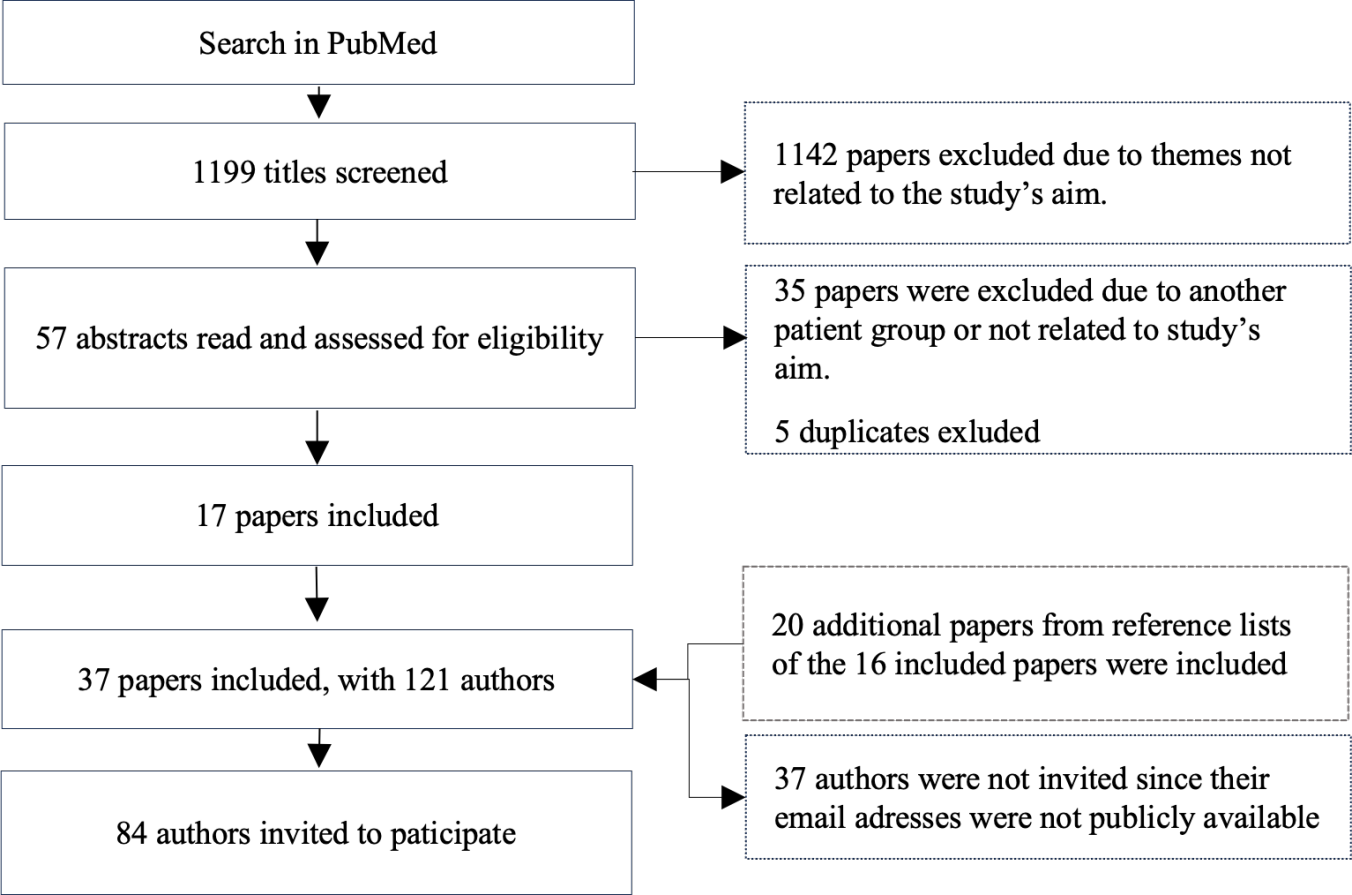
Flowchart for identification of scientific papers and participants for the Delphi study.

From the 1199 papers identified in the PubMed search and their relevant references, 37 scientific papers published between 2016 and 2023 were identified (Figure 1). The majority of the papers were from the United States (n=21), while 10 were from Europe, 4 were from Canada, and 1 was from Australia. Most papers used a quantitative method (n=14); 11 were qualitative, 4 were systematic and scoping reviews, and 7 were other studies such as mixed methods studies and a Delphi study (Multimedia Appendix 1). The identified papers had a total of 121 authors, of which email addresses were identified for 84 authors (Figure 1).

Among the 84 invited Delphi participants (ie, the authors of the identified papers), 27 (32%) responded in round 1; of whom, 21 (78%) responded in round 2. Most participants were researchers, and approximately one-third were health care professionals. Among the 7 health care professionals, 2 were exclusively health care professionals, while the remaining were also researchers. Most of the authors were female (Table 2).

**Table 2.** Characteristics of the Delphi participants.

		Round 1 (n=27), n (%)	Round 2 (n=21), n (%)
**Gender**			
	Female	22 (81)	17 (81)
	Male	5 (19)	4 (19)
	Other	0 (0)	0 (0)
**Age (years)**			
	31‐40	7 (26)	8 (38)
	41‐50	12 (44)	7 (33)
	51‐60	5 (19)	5 (24)
	61‐70	3 (11)	1 (5)
**Profession**			
	Researcher	23 (85)	18 (90)
	Health care provider	10 (37)	7 (35)
	Patient, informal caregiver, or user representative	2 (7)	2 (10)
	Student	1 (4)	0 (0)
	Other	1 (4)	1 (5)

#### Development and Adjustment of Recommendations

Initially, 43 suggestions for recommendations were formulated focusing on various aspects of the digital sharing of notes, including informing about digital access to notes, the forms of the notes that are being shared, training and support for professionals, and the possibility of withholding notes from the adolescents (Multimedia Appendix 2).

**Figure 2.**
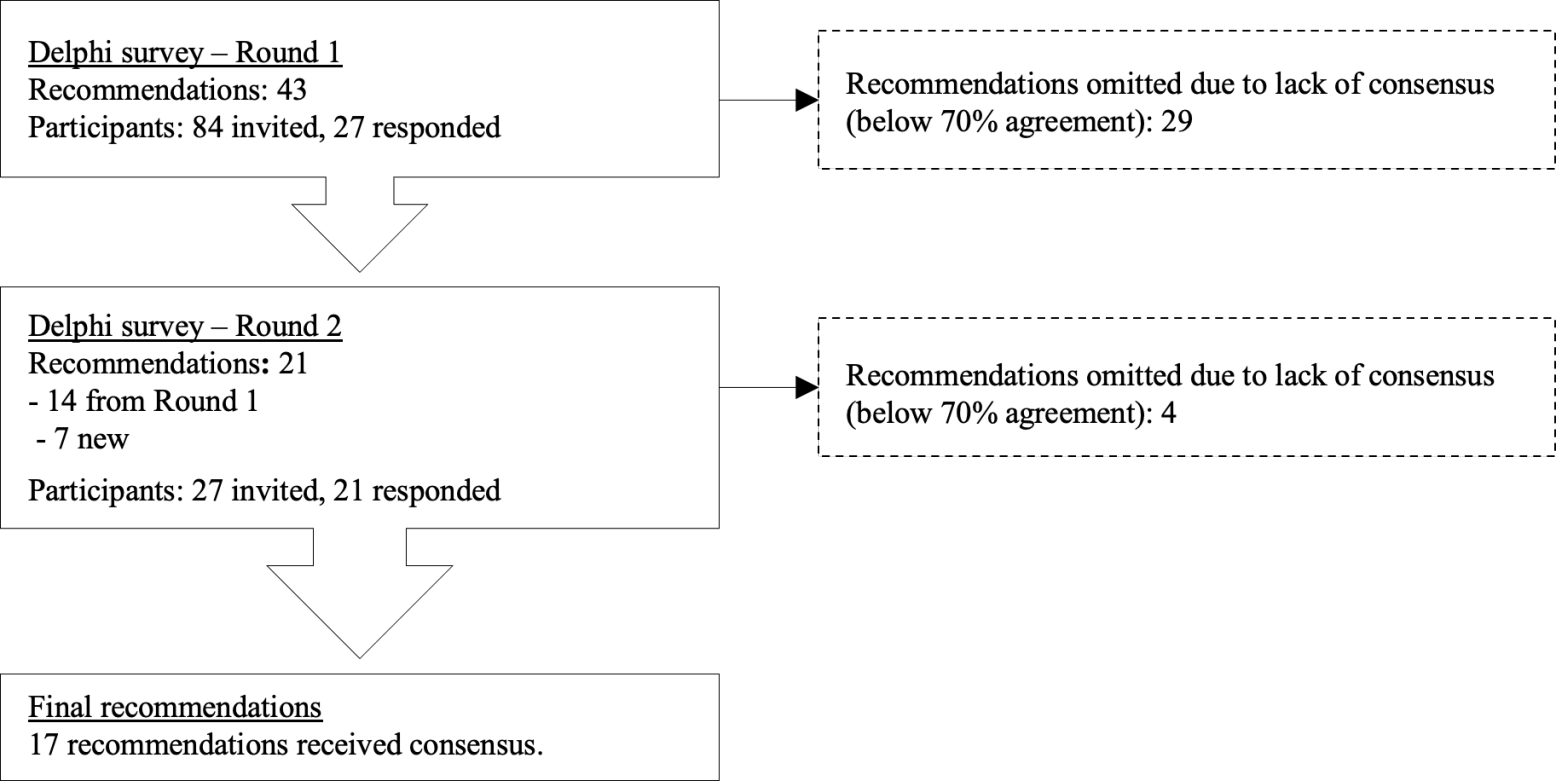
Flow diagram illustrating the two rounds of the Delphi study.

The responses from round 1 were analyzed for consensus (70% agreement), and the suggested recommendations that did not reach a consensus were dropped (Figure 2). Some recommendations were intentionally formulated to be mutually exclusive; thus, support for some was not anticipated. This included recommendations to abstain from any action, while others recommended specific actions. As a result, the Delphi participants were expected to support only one of these options.

The recommendations that did not reach a consensus in round 1 included mutually exclusive recommendations (n=9), at what age adolescents should receive digital access to their notes or be informed about their access (n=9), the timing of digitally sharing notes with parents or guardians (n=5), and some of the points on how to inform the adolescent about their digital access to their notes (n=3). Three recommendations regarding withholding notes from adolescents did not reach a consensus in round 1, yet comments on these were used to formulate 3 new recommendations for round 2 (Table 3).

**Table 3.** Recommendations sent out in the final round (round 2), with the proportion of agreement for each recommendation in rounds 1 and 2.

Recommendations		Participants who agreed, n (%)	
		Round 1 (n=27)	Round 2 (n=21)
**Information about digital access to mental health notes should be given:**			
	1a ...only if considered appropriate by the health care provider.^a^	19 (70)	5 (25)
	1b ...between the first contact with the service and the first clinical consultation.	—^b^	17 (81)
	1c ...when having a consultation with the adolescent for the first time.^a^	24 (88)	20 (95)
	1d ...at later occasions if the adolescent has not comprehended the information initially provided.	—	12 (57)
	1e ...if requested by the adolescent.^a^	23 (85)	19 (90)
**When informing the adolescent about digital access to mental health notes:**			
	2a ...information should be provided on where the adolescent can learn more.^a^	24 (88)	18 (86)
	2b ...the sensitive nature of the notes should be discussed with the adolescent (eg, that they should not uncritically share information on social media).^a^	22 (81)	19 (90)
	2c ...parents’ or guardians’ potential access should be discussed.^a^	25 (93)	20 (95)
	2d ...the adolescent should be encouraged to ask questions.^a^	26 (96)	20 (95)
**Mental health notes shared with both other health care professionals and adolescents:**			
	3a ...should be written in a respectful language.	21 (77)	19 (90)
	3b ...should primarily be written to be useful for adolescents (eg, in plain language and avoiding or explaining medical terms).^a^	19 (70)	10 (48)
	3c ...should primarily be written to be useful for other health care professionals (eg, by using objective descriptions and medical terms).^a^	22 (81)	16 (76)
**Training and/or support should be provided:**			
	4a ...on how to write mental health notes.^a^	23 (85)	19 (90)
	4b ...with information about the legal and/or formal regulations on digital access to mental health notes for adolescents.^a^	25 (93)	20 (95)
	4c ...on how to digitally share mental health notes with adolescents.^a^	20 (74)	20 (95)
	4d ...on how to demonstrate to adolescents how they can access their mental health notes digitally.	—	16 (76)
	4e ...with allocated time for discussion about the practice of sharing mental health notes for adolescents.^a^	20 (74)	14 (67)
	4f ...on the routines for withholding mental health notes from the adolescent.	—	18 (86)
**It should be possible to withhold notes from the adolescent:**			
	5a ...if it endangers the adolescent’s life or causes serious harm to their health.	—	21 (100)
	5b ...if it endangers the next of kin’s life or causes serious harm to their health.	—	20 (95)
	5c ...after having done a case-by-case assessment following explicitly stated criteria with a process of review by others.	—	19 (90)

a Statement is reformulated or modified based on comments from round 1.

b New in round 2.

#### The Final List of Recommendations

A consensus was reached on recommendations concerning how to introduce digital access to notes, write notes, and support health care professionals, and when to withhold notes (Multimedia Appendix 3). Additionally, a consensus was reached on areas where professionals should receive support and training, and on three situations where it should be possible to withhold notes from adolescents. A subgroup analysis was also conducted comparing round 2 responses between participants who were also health care professionals and those who were not, but this did not reveal any noteworthy differences.

### Results From the Cross-Sectional Study

The 17 recommendations that achieved a consensus in the Delphi study were sent to staff at 4 child and adolescent specialist mental health care clinics in Norway to assess whether they agreed with the recommendations.

A total of 41 staff members responded, 90% (n=37) of whom were female. In total, 80% (n=33) of the respondents currently worked as health care professionals, 10% (n=4) worked in management positions, and 10% (n=4) worked as administrative staff. The largest groups of health care professionals were psychologists (n=21, 51%), medical doctors (n=5, 12%), and clinical social workers (n=3, 7%). Most informants (n=29, 70%) had worked at a specialist mental health care clinic for at least 5 years.

At child and adolescent specialist mental health care clinics, 70% (n=29) or more of staff agreed on 14 recommendations, and 60% (n=25) or more agreed on all 17 (Multimedia Appendix 4). The recommendations that the fewest staff agreed on were recommendation 1b (that information about digital access to notes should be given between the first contact with the service and the first clinical consultation; n=24, 59% agreed), recommendation 1e (that it should be given if requested by the adolescent; n=25, 61% agreed), and recommendation 3c (that notes should primarily be written to be useful for other health care professionals; n=27, 66% agreed).

Almost all staff agreed that training should be provided on how to write notes to be shared with the adolescent (n=40, 98%) and about the legal or formal regulations on digital access to notes for adolescents (n=40, 98%). Additionally, staff agreed it should be possible to withhold notes from the adolescent if it endangers their life or causes serious harm to the health of the adolescent or their next of kin (n=40, 98%).

## Discussion

### Principal Findings

A consensus was reached on 17 recommendations regarding central areas related to the digital sharing of notes with adolescents in mental health care who are legally old enough to access their notes digitally. The recommendations considered how to introduce digital access to notes, write notes, and support health care professionals, and when to withhold notes. At child and adolescent specialist mental health care clinics, 60% or more of the 41 staff members agreed with the 17 recommendations.

The findings of this study contribute to the knowledge about which recommendations related to the digital sharing of notes with adolescents are agreed on by both the authors of scientific papers and staff at child and adolescent specialist mental health care clinics. While similar recommendations for the digital sharing of notes with adolescents or with adults in mental health care have not been identified, the current recommendations resonate with the advice given to health care professionals regarding communicating with young people in mental health care about online behavior [17] and for adolescents’ transition into adult care [14-16]. The similarities include initiating a conversation about the specific topic during an initial meeting [17] or at an early stage [14-16]; encouraging adolescents to ask questions [15-17]; giving advice on relevant legal regulations [14]; and addressing confidentiality, parents’ role, and privacy [15,16].

Most recommendations also aligned with results from prior studies on this subject identified in the literature search. For instance, several studies in adult mental health care have highlighted the significance of communicating with patients about their access to clinical notes [9,22], the importance of how clinical notes are written when they are shared [22-24,57], and consideration of situations where some information should not be shared with the patient [22]. In addition, the need to offer support and training for health care professionals in sharing notes with adolescents [12,25] and in mental health care [8,10,26,58] has been reported in previous studies. Although consistency with prior studies was anticipated since the recommendations were based on these studies, some of the recommendations also stemmed from areas characterized by contradictory research findings [8,10,26,58].

Areas where no consensus was reached may indicate opposing opinions in the field [8,10] and geographical differences in regulations and practices [1,27,28]. In this study, a consensus was not reached on when adolescents or parents should have access to notes. This is consistent with the findings of a scoping review about sharing EHR information with children, adolescents, and parents, which reported inconclusive results and complexity related to both manual adjustments for sharing and set age limits for automatic access [10]. Moreover, it aligns with the multifaceted experiences of health care professionals with experiences of sharing clinical notes with adolescents and parents [5,10,29,59-61]. Studies have reported that health care professionals appreciate how sharing clinical notes with adolescents and parents encouraged them to ask questions about what they read [59] and improved communication [10,60], but it also could pose a threat to the adolescents’ autonomy and confidentiality [5,29]. Furthermore, health care professionals face ethical challenges in preventing adolescents from accessing information that their parents have shared and from reading confidential information about their adolescent [6,10,61]. Such individual aspects can challenge set age limits for access while at the same time making decisions for manual adjustments a complex and time-consuming process.

Both authors of scientific papers and staff from child and adolescent specialist mental health care clinics agreed that notes should be written in respectful language. However, they were less certain about who the main receiver of the notes should be, with three-quarters of the authors and two-thirds of the staff agreeing that notes should primarily be written to be useful for health care professionals. This is interesting considering the increased focus on writing in a language that patients can understand, with the aim of, for example, improving their understanding of and engagement with their health and treatment [30,62,63]. This may indicate that we are still in the early stages of implementing this practice, and there are some challenges associated with the multiple audiences of the notes.

A previous study reported that mental health care professionals working with adults experienced an overall improvement in the quality of their notes even though they adapted their note writing to make it more understandable to the patient [31]. However, mental health care professionals in another study were concerned about the consequences of omitting clinically relevant information that was considered inappropriate for the patient to read [7]. A study found that health care professionals in psychiatry were more likely to perform off-the-record journaling and underreporting than those in somatic health care when sharing clinical notes with adult patients [64]. Although these studies focused on mental health care professionals working with adults, their diverse results align with this study’s finding that both the authors of scientific papers and the staff reported that health professionals should receive training on note writing.

### Implications

The recommendations developed in this study need to be tested, and future research should be conducted to assess the consequences of following the recommendations. However, health care professionals currently do not have recommendations that can be used in clinical practice. The implementation should be accompanied by training and support.

### Strengths and Limitations

To the best of our knowledge, this is the first study to create recommendations for the digital sharing of notes with adolescents in mental health care. A strength of the study was the systematic process of developing recommendations based on research in the field. Moreover, international authors in the field evaluated and agreed on these recommendations, including researchers and health care professionals. Additionally, the recommendations underwent external validation, as most recommendations were supported by staff working at child and adolescent specialist mental health care clinics.

One limitation of the study was that the response rate for the Delphi study was relatively low (32%). This means that it is unclear whether the participants in the Delphi study were a selected group or representative of the researchers in the field. A limitation might be that most of the invited participants were authors from studies in the United States and Europe. While this might reflect that the practice of digital access to notes is more developed and common in these regions, we might have missed experiences from other settings and cultures. Similarly, selection bias could be connected to staff recruitment at child and adolescent specialist mental health care clinics. The most likely consequence is that those with a special interest will reply. Still, it is not possible to know whether they have opinions regarding the recommendations that diverge from others.

Another possible limitation is the external validity of the recommendations since they were based on studies from the United States; fewer were from Europe, and no studies were from South America, Asia, or Africa. The results may not be generalizable to these areas.

### Conclusions

A total of 17 recommendations related to key aspects of health care professionals’ digital sharing of notes with adolescents in mental health care achieved consensus. Health care professionals can use these recommendations to guide their practice of sharing notes with adolescents in mental health care. However, the effects and experiences of following these recommendations should be tested in clinical practice.

## Supplementary material

10.2196/57965 Multimedia Appendix 1 Scientific papers used to make recommendations and identify Delphi participants.

10.2196/57965 Multimedia Appendix 2 The 43 statements to be ranked by the participants in round 1 of the Delphi study.

10.2196/57965 Multimedia Appendix 3 Recommendations for digitally sharing notes with adolescents in mental health care.

10.2196/57965 Multimedia Appendix 4 Recommendations for digitally sharing notes with adolescents in mental health care: agreement among authors and staff.

## Abbreviations

CREDES: Guidance on Conducting and Reporting Delphi Studies
EHR: electronic health record
STROBE: Strengthening the Reporting of Observational Studies in Epidemiology
