# Supplementary material for: Development of Recommendations for the Digital Sharing of Notes With Adolescents in Mental Health Care: Delphi Study
Source: JMIR Ment Health. 2024 Jun 6;11:e57965. doi: 10.2196/57965 (PMC11185290; doi:10.2196/57965)
Supplement: Multimedia Appendix 1 [file mental-v11-e57965-s001.doc]

**Appendix 1 – Development of recommendations for digital sharing of notes with adolescents in mental health care: a Delphi study**

**Scientific papers used to make recommendations and identify Delphi participants**

The search for papers was done in PubMed in April 2023 and by searching the references of the papers meeting the eligibility criteria. The following terms were used in the PubMed search: "(((Adolescent[MeSH Terms]) OR (Mental Health[MeSH Terms])) OR (Psychiatr*[MeSH Terms])) AND (electronic health record*[MeSH Terms])” published between 2021-2023.

**Included papers from the PubMed search:**

1. Nielsen MS, Steinsbekk A, Nøst TH. Views on patient portal use for adolescents in mental health care-a qualitative study. BMC Health Services Research. 2023;23(1): p. 1-9.
2. Fagerlund, A. J., Kristiansen, E., Simonsen, R. A. Experiences from using patient accessible electronic health records-a qualitative study within Sámi mental health patients in Norway. International Journal of Circumpolar Health, 2022; 81(1), 2025682.
3. Hagström J, Blease C, Haage B, Scandurra I, Hansson S, Hägglund M. Views, use, and experiences of web-based access to pediatric electronic health records for children, adolescents, and parents: scoping review. Journal of Medical Internet Research. 2022;24(11):e40328.
4. Zanaboni P, Kristiansen E, Lintvedt O, Wynn R, Johansen MA, Sørensen T, et al. Impact on patient-provider relationship and documentation practices when mental health patients access their electronic health records online: a qualitative study among health professionals in an outpatient setting. BMC Psychiatry. 2022;22(1): p. 508.
5. Hochwarter, S., Fehler, G., Muente, C., Eisenmann, Y., Heinze, M., Hibsch, C., et al. Design of a Patient-Accessible Electronic Health Record System in Mental Health. In Challenges of Trustable AI and Added-Value on Health. IOS Press. 2022; p. 583-584.
6. Bärkås A, Hägglund M, Moll J, Cajander Å, Rexhepi H, Hörhammer I, et al. Patients’ Access to Their Psychiatric Records–A Comparison of Four Countries. Challenges of Trustable AI and Added-Value on Health: IOS Press; 2022. p. 510-4.
7. Hagström J, Scandurra I, Moll J, Blease C, Haage B, Hörhammer I, et al. Minor and parental access to electronic health records: differences across four countries. Challenges of Trustable AI and Added-Value on Health: IOS Press; 2022. p. 495-9.
8. Kariotis, T. C., Prictor, M., Chang, S., Gray, K. Impact of electronic health records on information practices in mental health contexts: scoping review. Journal of Medical Internet Research. 2022;24(5), e30405.
9. Sipanoun, P., Oulton, K., Gibson, F., Wray, J.The experiences and perceptions of users of an electronic patient record system in a pediatric hospital setting: a systematic review. International journal of medical informatics. 2022;160, 104691.
10. Blease, C., Kharko, A., Hägglund, M., O’Neill, S., Wachenheim, D., Salmi, L., Torous, J. The benefits and harms of open notes in mental health: A Delphi survey of international experts. Plos one. 2021;16(10), e0258056.
11. Bruni, T., LaLonde, L., Maragakis, A., Lee, J., Caserta, A., Kilbourne, A. M., & Lancaster, B. The use of electronic health record tools to improve evidence-based treatment of adolescent depression in primary care. Academic Pediatrics. 2021;21(7), p. 1195-1202.
12. Blease, C. R., O'Neill, S. F., Torous, J., DesRoches, C. M., Hagglund, M. Patient access to mental health notes: motivating evidence-informed ethical guidelines. The Journal of nervous and mental disease. 2021;209(4), p. 265-269.
13. Pageler, N. M., Webber, E. C., Lund, D. P. Implications of the 21st Century Cures Act in pediatrics. Pediatrics. 2021;147(3).
14. Kelly, M. M., Smith, C. A., Hoonakker, P. L., Nacht, C. L., Dean, S. M., Sklansky, D. J., Coller, R. J. Stakeholder perspectives in anticipation of sharing physicians’ notes with parents of hospitalized children. Academic pediatrics. 2021;21(2), p. 259-264.
15. Dohil, I., Cruz, R., Sweet, H., Huang, J. S. Sharing Notes With Adolescents and Young Adults Admitted to an Inpatient Psychiatry Unit. Journal of the American Academy of Child and Adolescent PsychiatrY. 2021;60(3), p. 317-320.
16. van Rijt, A. M., Hulter, P., Weggelaar-Jansen, A. M., Ahaus, K., & Pluut, B. Mental health care Professionals’ appraisal of Patients’ use of web-based access to their electronic health record: qualitative study. Journal of Medical Internet Research. 2021;23(8), e28045.
17. Schwarz, J., Bärkås, A., Blease, C., Collins, L., Hägglund, M., Markham, S., Hochwarter, S. Sharing clinical notes and electronic health records with people affected by mental health conditions: scoping review. JMIR mental health. 2021;8(12), e34170.

**Scientific papers identified from references in the included papers from PubMed (published between 2016-2020):**

1. Goldstein, R. L., Anoshiravani, A., Svetaz, M. V., Carlson, J. L. Providers' perspectives on adolescent confidentiality and the electronic health record: a state of transition. Journal of Adolescent Health. 2020;66(3), p. 296-300.
2. Strudwick, G., Booth, R. G., McLean, D., Leung, K., Rossetti, S., McCann, M., et al. Identifying indicators of meaningful patient portal use by psychiatric populations. Informatics for Health and Social Care. 2020;45(4), p. 396-409.
3. Strudwick, G., Yeung, A., Gratzer, D. Easy access, difficult consequences? providing psychiatric patients with access to their health records electronically. Frontiers in Psychiatry. 2019:10, p. 917.
4. O’Neill, S., Chimowitz, H., Leveille, S., Walker, J. Embracing the new age of transparency: mental health patients reading their psychotherapy notes online. Journal of Mental Health. 2019;28(5), p. 527-535.
5. Miklin, D. J., Vangara, S. S., Delamater, A. M., Goodman, K. W. Understanding of and barriers to electronic health record patient portal access in a culturally diverse pediatric population. JMIR medical informatics. 2019;7(2), e11570.
6. Pisciotta, M., Denneson, L. M., Williams, H. B., Woods, S., Tuepker, A., Dobscha, S. K. Providing mental health care in the context of online mental health notes: advice from patients and mental health clinicians. Journal of mental health. 2019;28(1), p. 64-70.
7. Leung, K., Clark, C., Sakal, M., Friesen, M., Strudwick, G. Patient and Family Member Readiness, Needs, and Perceptions of a Mental Health Patient Portal: A Mixed Methods Study. In ITCH. 2019; p. 266-270.
8. Ancker, J. S., Sharko, M., Hong, M., Mitchell, H., Wilcox, L. Should parents see their teen’s medical record? Asking about the effect on adolescent–doctor communication changes attitudes. Journal of the American Medical Informatics Association. 2018;25(12), p. 1593-1599.
9. Ancker, J. S., Sharko, M., Hong, M., Mitchell, H., Wilcox, L. Should parents see their teen’s medical record? Asking about the effect on adolescent–doctor communication changes attitudes. Journal of the American Medical Informatics Association. 2018;25(12), p.1593-1599.
10. Klein, J. W., Peacock, S., Tsui, J. I., O’Neill, S. F., DesRoches, C. M., Elmore, J. G. Perceptions of primary care notes by patients with mental health diagnoses. The Annals of Family Medicine. 2018;16(4), p. 343-345.
11. Petersson, L., Erlingsdóttir, G. Open notes in Swedish psychiatric care (part 2): survey among psychiatric care professionals. JMIR mental health. 2018;5(2), e10521.
12. Denneson, L. M., Chen, J. I., Pisciotta, M., Tuepker, A., Dobscha, S. K. Patients’ positive and negative responses to reading mental health clinical notes online. Psychiatric Services.2018;69(5), p. 593-596.
13. Stablein, T., Loud, K. J., DiCapua, C., Anthony, D. L. The catch to confidentiality: the use of electronic health records in adolescent health care. Journal of Adolescent Health. 2018;62(5), p. 577-582.
14. Petersson, L., Erlingsdóttir, G. Open notes in Swedish psychiatric care (Part 1): survey among psychiatric care professionals. JMIR mental health. 2018;5(1), e9140.
15. Peck, P., Torous, J., Shanahan, M., Fossa, A., Greenberg, W. Patient access to electronic psychiatric records: a pilot study. Health policy and technology. 2017;6(3), p. 309-315.
16. Cromer, R., Denneson, L. M., Pisciotta, M., Williams, H., Woods, S., Dobscha, S. K. Trust in mental health clinicians among patients who access clinical notes online. Psychiatric Services. 2017;68(5), p. 520-523.
17. Denneson, L. M., Cromer, R., Williams, H. B., Pisciotta, M., Dobscha, S. K. A qualitative analysis of how online access to mental health notes is changing clinician perceptions of power and the therapeutic relationship. Journal of medical Internet research. 2017; 19(6), e208.
18. Kipping, S., Stuckey, M. I., Hernandez, A., Nguyen, T., Riahi, S. A web-based patient portal for mental health care: benefits evaluation. Journal of medical Internet research. 2016;18(11), e294.
19. Hong, M. K., Wilcox, L., Feustel, C., Wasileski-Masker, K., Olson, T. A., Simoneaux, S. F. Adolescent and caregiver use of a tethered personal health record system. In AMIA Annual Symposium Proceedings (Vol. 2016, p. 628). American Medical Informatics Association.
20. Thompson, L. A., Martinko, T., Budd, P., Mercado, R., & Schentrup, A. M. Meaningful use of

a confidential adolescent patient portal. Journal of Adolescent Health. 2016;58(2), p. 134-140.
