# Supplementary material for: Development of Recommendations for the Digital Sharing of Notes With Adolescents in Mental Health Care: Delphi Study
Source: JMIR Ment Health. 2024 Jun 6;11:e57965. doi: 10.2196/57965 (PMC11185290; doi:10.2196/57965)
Supplement: Multimedia Appendix 2 [file mental-v11-e57965-s002.doc]

**Appendix 2 – Development of recommendations for digital sharing of notes with adolescents in mental health care: a Delphi study**

**The 43 statements to be ranked by the participants in Round 1 of the Delphi study.**

| **Statements** | **Ranking from**  **1 – 5**  (1= least important, 5= most important) |
| --- | --- |
| **Adolescents should get digital access to their mental health notes** | |
| 1. From the age of 12 |  |
| 1. When adolescents are above the age of majority under health law. |  |
| 1. When adolescents express an interest themselves. |  |
| 1. When adolescents express an interest after being asked by a healthcare provider. |  |
| 1. When parents/guardians allow it after being asked by a healthcare provider. |  |
| 1. When parents/guardians allow it after being asked by a healthcare provider. |  |
| 1. When the adolescent does not have a serious mental illness. |  |
| 1. Never. |  |
| Comments to the statement above |  |
| **Parents/guardians should get digital access to the adolescent´s mental health notes:** | |
| 1. Until the adolescent turns 12. |  |
| 1. When the adolescent are below the age of majority under health law. |  |
| 1. When requested by the adolescent. |  |
| 1. When considered important by the healthcare provider based on case-by-case assessments. |  |
| 1. When the adolescent has consented to share. |  |
| 1. Never. |  |
| Comments to the statements above |  |
| **Healthcare providers should have the possibility to withhold mental health notes from the adolescent and their parents/guardians:** | |
| 1. After having completed a case-by-case assessment. |  |
| 1. After having discussed it with a colleague. |  |
| 1. After the clinic´s management has approved it. |  |
| 1. Never. |  |
| **Mental health notes to be shared with adolescents and parents/guardians should be written:** | |
| 1. In plain language. |  |
| 1. In a respectful language to avoid insulting or hurting the adolescent or parents/guardians. |  |
| 1. In a language that is useful for other healthcare providers. |  |
| Comments to the statements above. |  |
| **The healthcare provider should inform the adolescent about the formulation and content of the mental health notes during the consultation:** | |
| 1. To prepare the adolescent for what will be written in the mental health notes including diagnosis. |  |
| 1. To invite the adolescent to be involved in the note writing. |  |
| 1. Never. |  |
| Comments to the statements above. |  |
| **Healthcare providers should discuss the possibility of digitally accessing mental health notes with the adolescent:** | |
| 1. At every consultation. |  |
| 1. In the first consultation. |  |
| 1. When the adolescent is above the age of majority under health law. |  |
| 1. When considered appropriate by the healthcare providers. |  |
| 1. When requested by the adolescent |  |
| 1. When requested by the parents/guardians. |  |
| 1. Never |  |
| Comments to the statements above |  |
| **Healthcare providers should inform the adolescent about the possibility of digital access to mental health notes by:** | |
| 1. Providing information about where the adolescent can learn more. |  |
| 1. Giving a hands-on demonstration on how to access the mental health notes. |  |
| 1. Mentioning it briefly. |  |
| 1. Never. |  |
| Comments to the statements above |  |
| **If informing the adolescent about the possibility of digital access to mental health notes, the healthcare providers should:** | |
| 1. Talk about the sensitive nature of the mental health notes (e.g., that they should not uncritically share information on social media) |  |
| 1. Discuss parents’/guardians’ potential access. |  |
| 1. Encourage the adolescent to ask questions. |  |
| Comments to the statements above |  |
| **Healthcare providers should receive training on sharing mental health notes digitally in adolescent mental health care:** | |
| 1. On how to write mental health notes when adolescents and parents/guardians have access. |  |
| 1. About the regional/national regulations on access for adolescents and their parents/guardians. |  |
| 1. On the technical part of how to digitally share mental health notes with adolescents and parents/guardians. |  |
| 1. During allocated time for discussion about note writing and sharing facilitated and supported by the management. |  |
| 1. Never |  |
| Comments to the statements above. |  |
