# Supplementary material for: Development of Recommendations for the Digital Sharing of Notes With Adolescents in Mental Health Care: Delphi Study
Source: JMIR Ment Health. 2024 Jun 6;11:e57965. doi: 10.2196/57965 (PMC11185290; doi:10.2196/57965)
Supplement: Multimedia Appendix 3 [file mental-v11-e57965-s003.doc]

**Appendix 3 – Development of recommendations for digital sharing of notes with adolescents in mental health care: a Delphi study**

**Recommendations for digital sharing of notes with adolescents in mental health care**

***Information about digital access to mental health notes should be given (…):***

- Between the first contact with the service and the first clinical consultation.
- When having a consultation with the adolescent for the first time.
- If requested by the adolescent.

***When informing the adolescent about digital access to mental health notes (…):***

- Information should be provided on where the adolescent can learn more.
- The sensitive nature of the notes should be discussed with the adolescent (e.g., that they should not uncritically share information on social media).
- Parents’ or guardians’ potential access should be discussed.
- The adolescent should be encouraged to ask questions.

***Mental health notes shared with both other healthcare professionals and adolescents (…):***

- Should be written in a respectful language.
- Should primarily be written to be useful for other healthcare providers (e.g., by using objective descriptions and medical terms).

***Training and/or support should be provided (…):***

- On how to write mental health notes.
- With information about the legal and/or formal regulations on digital access to mental health notes for adolescents.
- On how to digitally share mental health notes with adolescents.
- On how to demonstrate to adolescents how they can access their mental health notes digitally.
- On the routines for when withholding mental health notes from the adolescent.

***It should be possible to withhold notes from the adolescent (…):***

- If it endangers the adolescent’s life or causes serious harm to their health.
- If it endangers the next of kin's life or causes serious harm to their health.
- After having done a case-by-case assessment following explicitly stated criteria with a process of review by others.
