# Supplementary material for: Development of Recommendations for the Digital Sharing of Notes With Adolescents in Mental Health Care: Delphi Study
Source: JMIR Ment Health. 2024 Jun 6;11:e57965. doi: 10.2196/57965 (PMC11185290; doi:10.2196/57965)
Supplement: Multimedia Appendix 4 [file mental-v11-e57965-s004.doc]

**Appendix 4 – Development of recommendations for digital sharing of notes with adolescents in mental health care: a Delphi study**

**Recommendations for digital sharing of notes with adolescents in mental health care – agreement among authors and staff.**

| ***Information about digital access to mental health notes should be given (…):*** | ***% agreement authors*** | ***% agreement staff*** |
| --- | --- | --- |
| - between the first contact with the service and the first clinical consultation. | 81 % | 59 % |
| - when having a consultation with the adolescent for the first time. | 95 % | 88 % |
| - if requested by the adolescent. | 90 % | 61 % |
| ***When informing the adolescent about digital access to mental health notes (…):*** |  |  |
| - information should be provided on where the adolescent can learn more. | 86 % | 78 % |
| - the sensitive nature of the notes should be discussed with the adolescent (e.g., that they should not uncritically share information on social media). | 90 % | 95 % |
| - parents’ or guardians’ potential access should be discussed. | 95 % | 88 % |
| - the adolescent should be encouraged to ask questions. | 95 % | 73 % |
| ***Mental health notes shared with both other healthcare professionals and adolescents (…):*** |  |  |
| - should be written in a respectful language. | 90 % | 95 % |
| - should primarily be written to be useful for other healthcare providers (e.g., by using objective descriptions and medical terms). | 76 % | 66 % |
| ***Training and/or support should be provided (…):*** |  |  |
| - on how to write mental health notes. | 90 % | 98 % |
| - with information about the legal and/or formal regulations on digital access to mental health notes for adolescents. | 95 % | 98 % |
| - on how to digitally share mental health notes with adolescents. | 95 % | 90 % |
| - on how to demonstrate to the adolescents how they can access their mental health notes digitally. | 76 % | 73 % |
| - on the routines for when withholding mental health notes from the adolescent. | 86 % | 95 % |
| ***It should be possible to withhold notes from the adolescent (…):*** |  |  |
| - if it endangers the adolescent’s life or causes serious harm to their health. | 100 % | 98 % |
| - if it endangers the next of kin's life or causes serious harm to their health. | 95 % | 98 % |
| - after having done a case-by-case assessment following explicitly stated criteria with a process of review by others. | 90 % | 95 % |
